# Doxycycline is an NF-κB inhibitor that induces apoptotic cell death in malignant T-cells

**DOI:** 10.18632/oncotarget.12488

**Published:** 2016-10-06

**Authors:** Carolina V. Alexander-Savino, Matthew S. Hayden, Christopher Richardson, Jiyong Zhao, Brian Poligone

**Affiliations:** ^1^ Rochester General Hospital Research Institute, Center for Cancer and Blood Disorders, Rochester, NY, USA; ^2^ Division of Allergy, Immunology and Rheumatology, University of Rochester School of Medicine, Rochester, NY, USA; ^3^ Department of Biomedical Genetics, University of Rochester School of Medicine, Rochester, NY, USA; ^4^ Rochester Skin Lymphoma Center, Fairport, NY, USA

**Keywords:** non-Hodgkin's lymphoma, signal transduction, apoptosis, drug repurposing, doxycycline

## Abstract

Cutaneous T-cell Lymphoma (CTCL) is a rare non-Hodgkin's lymphoma that can affect the skin, blood, and lymph nodes, and can metastasize at late stages. Novel therapies that target all affected disease compartments and provide longer lasting responses while being safe are needed. One potential therapeutic target is NF-λB, a regulator of immune responses and an important participant in carcinogenesis and cancer progression. As a transcription factor, NF-λB targets genes that promote cell proliferation and survival. Constitutive or aberrant activation of NF-λB is encountered in many types of cancer, including CTCL.

Recently, while analyzing gene-expression profiles of a variety of small molecule compounds that target NF-λB, we discovered the tetracycline family of antibiotics, including doxycycline, to be potent inhibitors of the NF-λB pathway. Doxycycline is well-tolerated, safe, and inexpensive; and is commonly used as an antibiotic and anti-inflammatory for the treatment a multitude of medical conditions.

In our current study, we show that doxycycline induces apoptosis in a dose dependent manner in multiple different cell lines from patients with the two most common subtypes of CTCL, Mycosis Fungoides (MF) and Sézary Syndrome (SS). Similar results were found using primary CD4+ T cells from a patient with SS. Doxycycline inhibits TNF induced NF-λB activation and reduces expression of NF-λB dependent anti-apoptotic proteins, such as BCL2α. Furthermore, we have identified that doxycycline induces apoptosis through reactive oxygen species.

## INTRODUCTION

Cutaneous T-cell Lymphomas (CTCL) are rare forms of non-Hodgkin's lymphomas that encompass about half of all mature T-cell malignancies and primarily involve the skin [1]. Mycosis fungoides (MF) and Sézary Syndrome (SS) encompass about half of all CTCLs. MF is characterized by the presence of patches, plaques, or tumors, and in more advanced stages, it can present with erythroderma, affect the blood and lymph nodes, and metastasize to other organs [2]. Both MF and SS may be associated with severe pruritus that often reduces quality of life [2]. Although the median survival can vary from 10-35 years in those with early stage disease, this drastically reduces to only 1.5 years for those with advanced staged disease [3]. Additionally, one in four people with early stage disease progress to more advanced stages with a drastic reduction in overall survival rate to 4 years [4, 5].

The heterogeneity in the histological and clinical presentations of CTCL presents a challenge to clinicians despite recent advances in treatment options for this disease [2, 6, 7]. There is an unmet need for therapies that can be effective in all disease compartments while being safe [2, 7]. There are currently few systemic therapies available, and these have overall response rates of 20-45% and complete response rates of less than 20%. The highest median duration of response is 15 months [8–11]. Many of these patients do not respond to standard chemotherapy [12]. Moreover, the toxicities of these therapies are often significant. Therefore, new therapies that are effective, safe, and tolerable are needed [13].

The “Connectivity Map” is a research effort of the Broad Institute that links gene expression patterns produced after treatment with over 1,300 small-molecule compounds [14]. This map facilitates the discovery of new drugs for the treatment of diseases by allowing researchers to screen for compounds that could target genes known to be involved in pathways of disease, such as those controlled by transcription factor NF-κB. NF-κB regulates genes that promote cell proliferation, metastasis, angiogenesis, invasion, inflammation, and cell survival. NF-κB is a key regulator of immune response and important in carcinogenesis and cancer progression [15]. Constitutive expression of NF-κB is associated with a multitude of cancers, including CTCL [16–19]. We previously reported our effort to identify novel targets of the NF-κB pathway using the Connectivity map and doxycycline was identified as a novel inhibitor of this pathway [20].

Doxycycline is an inexpensive and widely used tetracycline, commonly known for its antibiotic properties, that was first synthesized from chlortetracycline in 1967 [21]. Chlortetracycline is the parent structure for all tetracyclines and is naturally found in S*treptomyces aureofaciens* [22]. Doxycycline's antibiotic effects come from its ability to bind to the bacterial ribosome's 30s subunit and inhibit protein synthesis. This makes it capable of treating both gram positive and gram negative bacterial infections.

Independent from its antimicrobial activities, doxycycline has a vast range of pharmacological properties, including its ability to suppress inflammation due to inhibition of metalloproteinases, hydrolases, and cytokine production [23, 24]. Thus, it has been utilized for the treatment of various conditions, including cardiovascular and neurological disorders, periodontal disease, malaria, rickettsial and chlamydial infections, Lyme disease, and inflammatory dermatologic disorders such as rosacea, acne vulgaris, and bullous pemphigoid [25–27]. Doxycycline is usually well tolerated and has a low side-effect profile [28]. These therapeutic benefits, along with its inhibitory effect on NF-κB target genes, and its commercial availability, significantly reduces the amount of time it would take to bring a drug to the market, which prompted us to study the effects of doxycycline in cells from patients with CTCL.

## RESULTS

### Doxycycline treatment of CTCL cell lines leads to cell death

**Figure 1:**
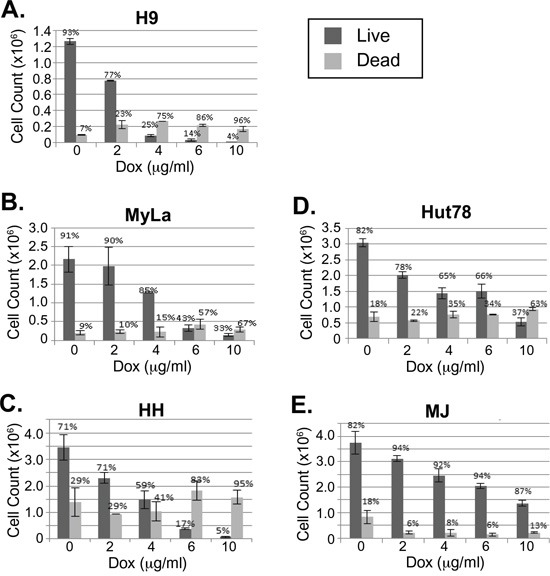
Doxycycline decreases viability of a subset of CTCL cell lines **A-E.** CTCL cell lines H9, MyLa, HH, Hut78, and MJ were cultured in the presence or absence of doxycycline (Dox) at the indicated concentrations. After four days of treatment, cell numbers and viability were assessed by light microscopy after staining with trypan blue and total live and dead cell counts were plotted. Error bars represent the standard deviation of duplicate or triplicate measurements.

Doxycycline was added at varying concentrations to actively growing cell lines H9, Hut78, HH, MyLa, and MJ. Viability was assessed using trypan blue after 4 days of treatment (Figure 1A–1E). Most of the cell lines, including H9, HH, Hut78, and MyLa, were killed by doxycycline in a dose dependent manner. However, MJ showed more resistance to doxycycline killing. Although there was some cell cycle arrest observed in the MJ cells at higher concentrations of doxycycline, even after 7 days of treatment, at the highest dose of doxycycline, MJ cells were still viable compared to the sensitive cell lines that showed essentially 100% killing after 4 days (data not shown).

**Figure 2:**
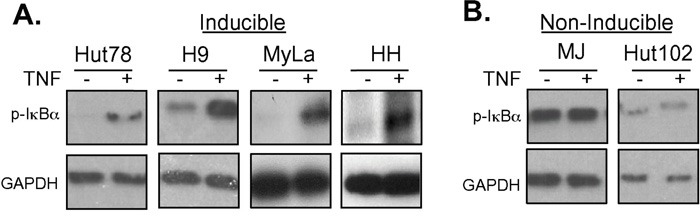
Doxycycline resistant cell lines are resistant to TNF-induced NF-κB pathway activation **A.** Cell lines that were sensitive to Dox treatment (Hut78, H9, MyLa and HH) or **B.** those that were insensitive to Dox treatment (MJ and Hut102) were treated with TNF for 5 or 15 minutes. Whole cell lysates were prepared and resolved in 10% SDS PAGE gels, electroblotted onto PDVF membranes, and subjected to Western blotting using antibodies specific for p-IκBα and GAPDH (loading control).

These results were interesting as they correlated with results that were discovered while examining NF-κB activity in these CTCL cell lines. IκBα keeps NF-κB sequestered in the cytoplasm, thereby preventing it from performing its function as a transcription factor in the nucleus. TNFα (TNF) induces phosphorylation of IκBα, triggering its ubiquitination and subsequent degradation in the proteasome. This frees NF-κB in the cytoplasm to migrate into the nucleus and transcribe genes that are crucial for cell survival and proliferation [29–31]. Interestingly, the sensitivity to doxycycline killing (Figure 1A–1E) correlated with the cell line's ability to induce NF-κB (Figure 2A–2B). TNF treatment increased NF-κB pathway activation in Hut78, H9, MyLa, and HH cell lines (Figure 2A). In contrast, despite changes in baseline NF-κB pathway activation, we did not observe increased phosphorylation of IκBα in either MJ or Hut102 cells after TNF stimulation (Figure 2B). This suggests that CTCLs may have multiple mechanisms through which NF-κB is activated, as is known to occur in Activated B-cell like (ABC) and Germinal Center B-like (GBC) Diffuse Large B-Cell Lymphomas [16, 32]. Interestingly, of the CTCL cell lines studied, both MJ and Hut102 are known to be HTLV positive while the others (Hut78, H9, MyLa, and HH), and, in fact most primary CTCLs, are known to be HTLV negative. This is of particular interest given that HTLV Tax has pleiotropic effects on the NF-κB pathway and is well known to activate both canonical and non-canonical NF-κB pathways [33].

### Doxycycline inhibits NF-κB in CTCL cell lines

**Figure 3:**
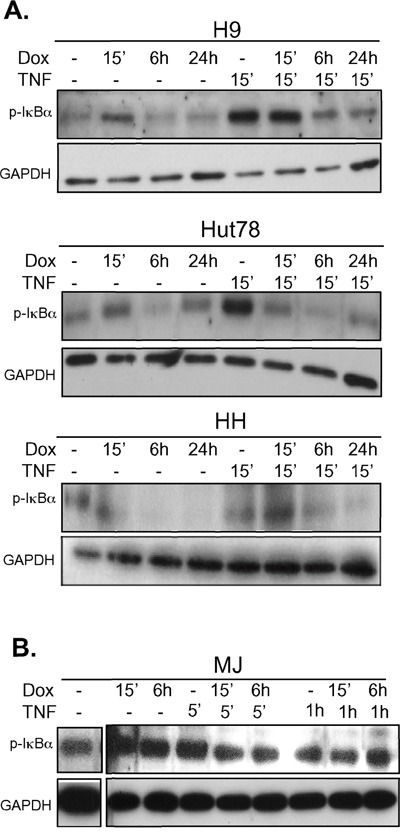
Doxycycline pre-treatment prevents TNF induced phosphorylation of IκBα in doxycycline-sensitive CTCL cell lines **A.** CTCL cell lines that are sensitive or **B.** those that were insensitive to doxycycline were assayed for the effects of doxycycline on TNF signaling to NF-κB. The indicated cell lines were either untreated or pre-treated with doxycycline (Dox, 5μg/mL) for the indicated period of time up to 24h. Cells were then either left untreated or treated with TNF as indicated. Whole cell lysates were prepared and resolved in 10% SDS PAGE gels, electroblotted onto PDVF membranes, and protein levels of p-IκBα and GAPDH (loading control) were assessed using specific antibodies.

We have previously shown that doxycycline is an inhibitor of NF-κB in some cell lines [20]. Given that doxycycline induces cellular killing in CTCL cell lines that also can be further activated with TNF, we next examined if doxycycline could inhibit TNF-mediated NF-κB activation in these cells. Cells were pretreated with doxycycline for various times and then stimulated with TNF for various times. The amount of phosphorylated IκBα was assessed by western blotting. Cell lines that were sensitive to doxycycline treatment (H9, MyLa, Hut78, and HH) (Figure 1A–1D), in which NF-κB was induced after TNF stimulation (Figure 2A), showed decreased levels of p-IκBα after TNF stimulation in the presence of doxycycline (Figure 3A and data not shown). In contrast, the cell lines that were HTLV-1 positive, had constitutively high levels of p-IκBα that were not further inducible by TNF administration (Figure 2B), showed no effect on p-IκBα levels after TNF stimulation with doxycycline pretreatment (Figure 3B). Therefore, the cell lines that were most sensitive to doxycycline killing were those in which NF-κB was inducible with TNF stimulation. Most interestingly, we show that doxycycline can block NF-κB by inhibiting the TNF induced phosphorylation of IκBα. (Figure 3A).

### Doxycycline treatment of CTCL cell lines leads to apoptotic cell death

**Figure 4:**
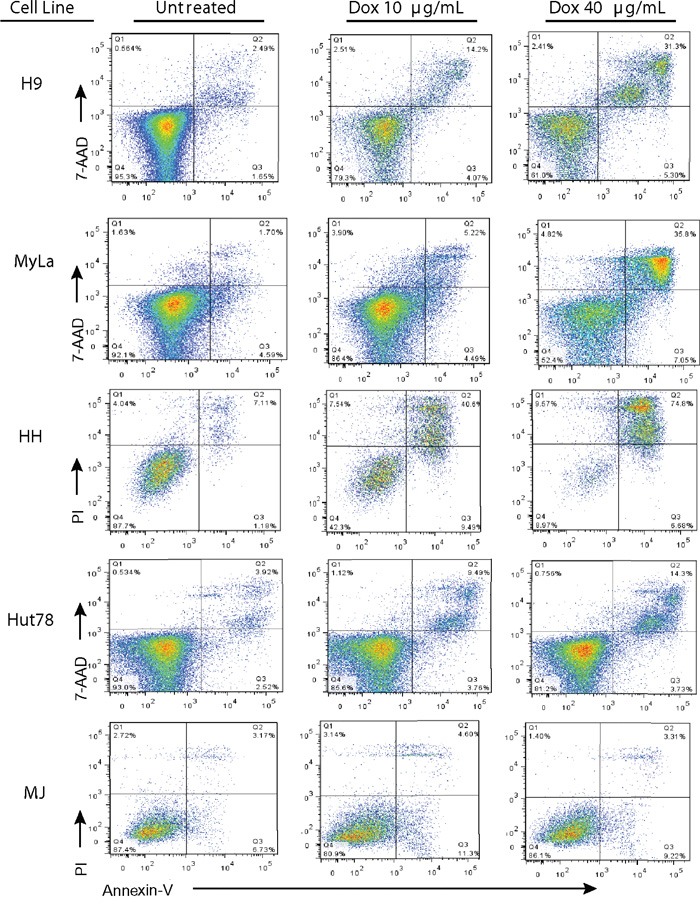
Doxycycline induces programmed cell death in sensitive CTCL cell lines Doxycycline sensitive (H9, MyLa, HH, and Hut78) and insensitive (MJ) CTCL cell lines were either untreated or treated with the specified concentrations of Doxycycline (10μg/mL or 40 μg/mL) for 48 hours (H9, MyLa, and HH) or 96 hours (Hut78 and MJ). Treated cells were then stained with PI or 7AAD and PE-Annexin V, and analyzed by flow cytometry.

To determine if dying cells were undergoing programmed cell death after doxycycline treatment, cells were treated with doxycycline and stained with Annexin V and either 7AAD or propidium iodide (PI). Live and dead cells were plotted against Annexin V stains to detect which ones had undergone programmed cell death (Figure 4). Similar to the trypan blue exclusion assay results (Figure 1), doxycycline increased the percentage of late apoptotic cells in a dose dependent manner in H9, MyLa, HH, and Hut78 CTCL cell lines, while MJ cells were resistant. For example, the percentage of late apoptotic H9 cells increased with increasing doxycycline concentrations, from 2.49% (untreated) to 14.2% (10 μg/mL) and 31.3% (40 μg/mL). Moreover, MJ cells showed little Annexin V positive dead cells regardless of the doxycycline concentration used.

### The extrinsic apoptotic pathway is important in doxycycline killing

We next examined classic signatures of apoptosis. There are two main pathways of apoptosis, which differ in the way that initiator caspases are activated. The extrinsic pathway is activated through death receptors and the intrinsic pathway acts through the mitochondria [34]. These pathways may act separately or together depending on cell type but eventually converge when effector caspases are activated [35]. This leads to protein cleavage, DNA fragmentation, membrane blebbing, and the formation of apoptotic bodies, which are signature characteristics of apoptosis [36].

**Figure 5:**
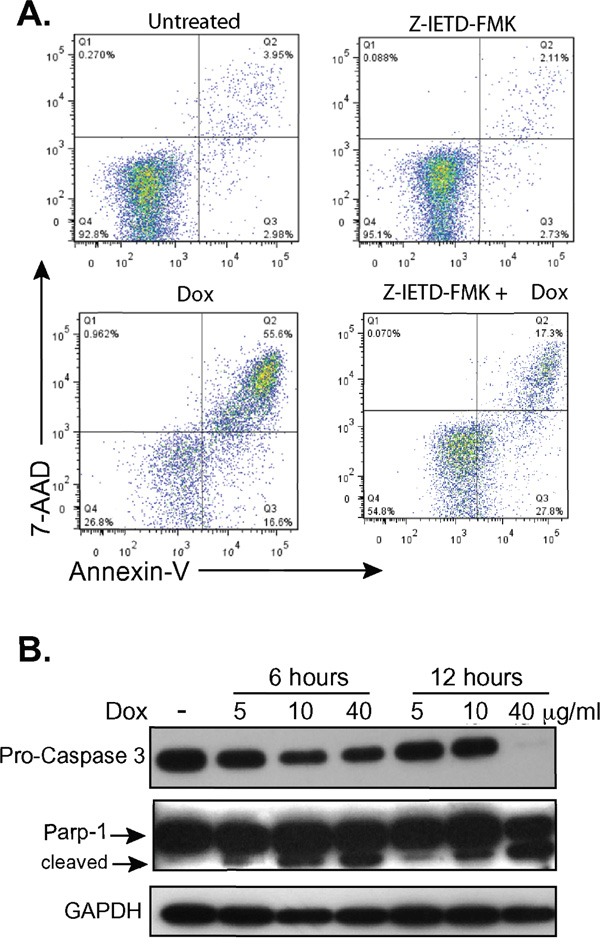
Doxycycline affects the extrinsic pathway of apoptosis **A.** H9 cells were treated with or without doxycycline (Dox, 10μg/mL) and/or the caspase-8 inhibitor Z-IETD-FMK for 48 hours, stained with 7AAD and PE-Annexin V, and analyzed by flow cytometry. **B.** Western blots of whole cell lysates from H9 cells treated with doxycycline (Dox) at specified concentrations and timepoints show decreasing levels of pro-caspase 3 and formation of the 89KDa fragment of PARP-1 (cleaved). GAPDH levels were assessed as a loading control.

Caspase-8 initiates the extrinsic pathway upon death receptor ligand ligation and activates downstream effector caspases such as caspase-3 [37]. Caspase-3 activation leads to cleavage of PARP-1, a nuclear enzyme involved in DNA damage repair, which is fragmented into multiple catalytic components during apoptosis including the 24KDa and 89KDa fragments [37–39]. H9 cells treated with doxycycline demonstrated decreased levels of procaspase-3 and increased cleavage of PARP-1 in a dose dependent manner (Figure 5A), suggesting a potential role for caspase-8. To further evaluate if caspase-8 had a role in the programmed cell death observed in CTCL cells upon treatment with doxycycline, we treated H9 cells with the caspase-8 inhibitor, Z-IETD-FMK (Figure 5B). While late apoptosis was observed in 55.63% of cells treated with doxycycline alone, this decreased to 17.3% in cells additionally treated with the caspase 8 inhibitor. Collectively, these results show that one mechanism by which doxycycline induces apoptosis in CTCL cell lines is through caspase-8, an initiator to the extrinsic pathway of apoptosis.

### The intrinsic apoptotic pathway is important in doxycycline killing

**Figure 6:**
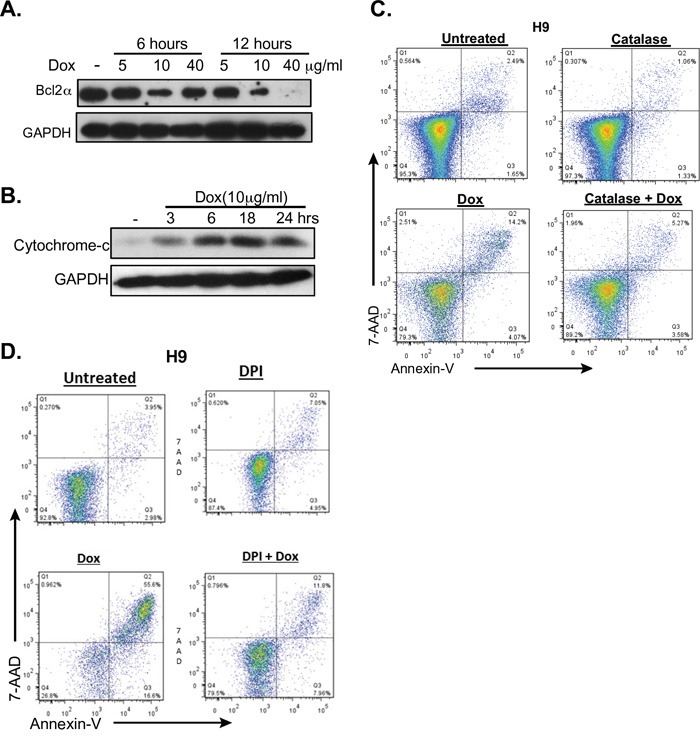
Doxycycline affects the intrinsic pathway of apoptosis **A.** and **B.** H9 cells were either untreated or treated with doxycycline (Dox) at the specified times and concentrations. A. Western blot analysis of cytoplasmic extracts for the NF-κB dependent, anti-apoptotic protein BCL2α. B. Cytoplasmic levels of cytochrome C were assessed by Western blotting. **C.** and **D.** Reversal of doxycycline induced apoptosis by ROS inhibition was assessed. H9 cells were untreated or treated with 10μg/ml doxycycline for 48 hours and apoptosis was assessed by flow cytometric analysis of 7AAD and PI staining. The effects of ROS inhibition were assessed by co-treating with either C. catalase, or D. diphenyleneiodonium chloride (DPI).

BCL2α prevents the production of Reactive Oxygen Species (ROS) at high levels within cells by inhibiting the release of cytochrome c from the mitochondria [40]. Moreover, BCL2a is an NF-κB dependent gene [41] that has been shown to be expressed in CTCLs [42–44]. Doxycycline treatment of CTCL cell lines correlated with decreased levels of BCL2α in a dose dependent manner (Figure 6A). This also correlated with increasing levels of cytochrome c in the cytoplasm (Figure 6B). These results suggest involvement of the intrinsic pathway of apoptosis in cells treated with doxycycline, likely due to increased levels of ROS.

### Apoptosis induced by doxycycline is due to reactive oxygen species

Studies have shown that stimulation of NF-κB by TNF activates the anti-oxidative defense in T-cells, and that inhibition of this pathway results in excess production of reactive oxygen species [45, 46]. Additionally, ROS have been shown to induce apoptosis in CTCL cells [47]. Furthermore, a recent study proposed that a global phenotype that is conserved among cancer cells is the dependence on mitochondrial biogenesis for clonal expansion and survival [48]. This study showed that several classes of FDA-approved antibiotics, including tetracyclines, can be used to eradicate cancer stem cells because they target the mitochondria. Mitochondria are the primary site for the production of ROS within cells and the resulting oxidative stress plays a very important role in apoptosis [49]. Due to the fact that our data suggested that ROS was important in apoptotic death induced by doxycycline in CTCL cells (Figure 6A and 6B), we next examined if inhibition of ROS could rescue cells from apoptosis. H9 and MyLa were incubated with catalase or flavoprotein inhibitor diphenyleneiodonium (DPI), both of which block mitochondria-derived ROS production [50]. Catalase catalyzes the breakdown of hydrogen peroxide into oxygen and water. As shown in Figure 6C and 6D, catalase and DPI significantly reduced the killing effect of doxycycline. In summary, our results indicate that doxycycline induced apoptosis is mediated through both the intrinsic and extrinsic pathways of apoptosis.

### Doxycycline kills primary malignant T-cells from a patient with Sézary Syndrome

**Figure 7:**
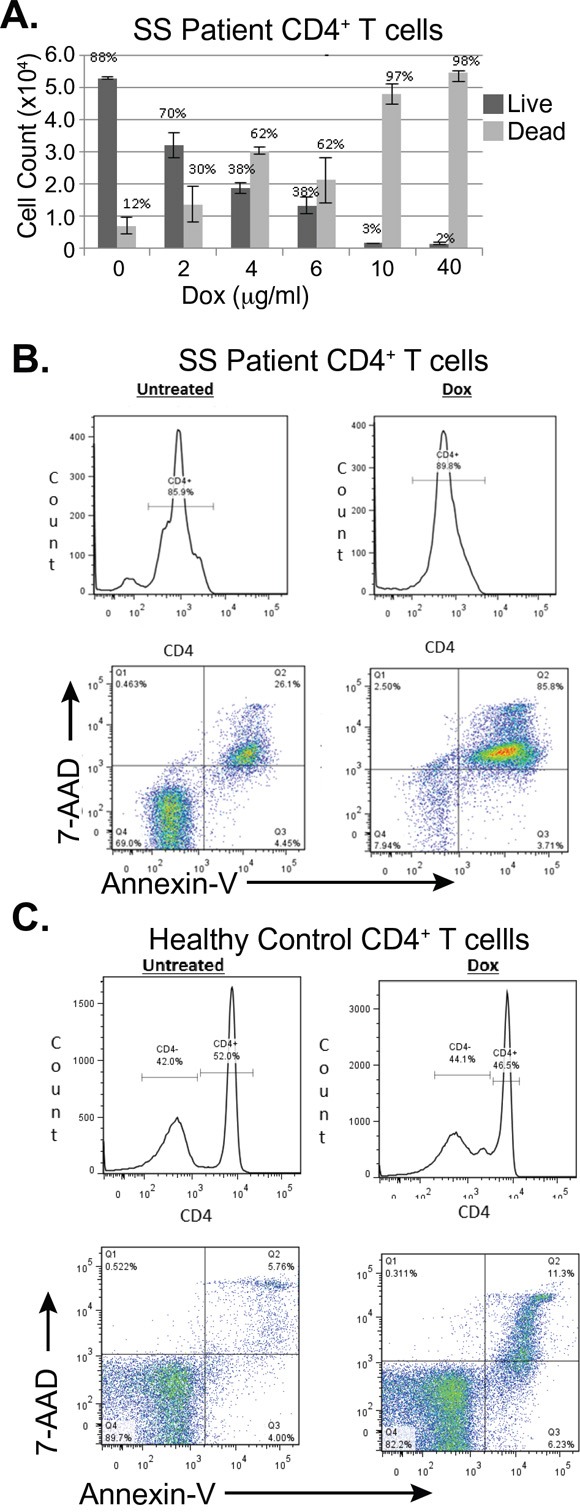
Doxycycline induces programmed cell death in primary CD4+ T-cells from a subject with Sézary Syndrome but not in those from a healthy subject **A.** Total CD4^+^ T cells from a patient with SS were treated *ex vivo* with the indicated concentrations of doxycycline (Dox) for 96 hours. Cell Viability was assessed using Trypan blue exclusion and total numbers of live and dead cells were plotted. Error bars represent the standard deviation of triplicate measurements. **B. C.** Flow cytometry was used to assess cell death in B. CD4+ primary Sézary Cells B. and CD4+ healthy cells C., which were stained with FITC-CD4, 7AAD and PE-Annexin V after either being untreated or treated with doxycycline (Dox, 40μg/mL) for 48 hours.

To see if doxycycline would induce apoptosis in primary cells, CD4+ T cells from a patient with Sézary Syndrome were treated with doxycycline. The patient was a 68 year old male, who presented with erythroderma. His flow cytometry showed that 65.72% of all lymphocytes were CD4+CD26-, 10.95% were CD4+CD7-, and that the CD4+/CD8+ ratio was 24.33. Trypan blue exclusion assays showed that CD4+ T-cells from this patient were killed by doxycycline in a dose dependent manner (Figure 7A). Additionally, after 48 hours of treatment with doxycycline 40μg/mL, 26.1% of the untreated cells were late apoptotic, while 85.8% of cells treated with doxycycline had died through apoptosis (Figure 7B). This was consistent over several blood draws from different dates. (Data not shown) A second subject with SS also showed decreased CD4+ T-cell viability upon doxycycline treatment (results not shown). Lastly, blood samples from a healthy individual showed that only 11.3% of T-cells underwent apoptosis after 48 hours of doxycycline treatment, which was similar to untreated control (Figure 7C).

## DISCUSSION

Although doxycycline induces apoptosis in most CTCL cell lines examined, two subgroups respond to treatment differently, and this response correlated with doxycycline's ability to inhibit phosphorylation of IκBα. The group that was sensitive to TNFα stimulation responded to doxycycline treatment, whereas the group that was insensitive, remained resistant. The fact that some cells could not be induced with TNF could explain why some CTCL patients might respond to therapies that inhibit NF-κB, such as bortezomib, while others do not. Indeed, previous studies have found a wide range of sensitivity of CTCL cells to NF-κB inhibition [47].

It is important to note that in our study those cells that were insensitive to TNFα stimulation and resistant to doxycycline, MJ and Hut102 cells, were HTLV-1 infected. HTLV-1 induces constitutive activation of NF-κB through Tax [33]. Tax is a viral protein that binds directly to the regulatory subunit of the kinase that phosphorylates IκBα, IKK. Thereafter, IKK constitutively activates IKKα and IKKβ causing degradation of IκBs, driving activation of both classical and alternative NF-κB pathways [51]. Furthermore, HTLV Tax also exerts multiple effects on NF-κB-dependent and –independent transcriptional events in infected cells. Given that MJ and Hut102 cell lines were insensitive to doxycycline, we speculate that doxycycline is unable to fully repress NF-κB, as well as other Tax-induced transcriptional events. However, given that in patients, CTCL cells are rarely infected with HTLV-1, it is unclear that this finding would significantly impact the potential clinical utility of doxycycline in CTCL treatment.

Consistent with these findings, our results show that doxycycline can target both Mycosis Fungoides and Sézary Syndrome cells. Doxycycline induces cell death through the activation of caspase-8 and the release of cytochrome c, suggesting the involvement of both the extracellular and intracellular pathways of apoptosis. Furthermore, doxycycline's ability to induce apoptosis in CTCL cells can be reversed through the inhibition of ROS. Treatment with oxidants has been shown to be sufficient when treating inflammatory conditions in which NF-κB is constitutively active [52]. Through the inhibition of NF-κB, as shown by inhibition of IκBα phosphorylation and decreased BCL2α levels, doxycycline increased ROS in CTCL cells and triggered apoptosis that could be reversed through treatment with antioxidants such as catalase and DPI.

Our results are interesting in light of a recent case study of a 21 year old female with a 5 year history of CTCL, primary cutaneous small/medium, pleomorphic CD4+ T cell lymphoma, who achieved a 13 month complete remission through oral treatment with doxycycline monohydrate at a dose of 200mg per day [53]. Moreover, the idea that antibiotics could be used to target a conserved weak point among different cancers was recently suggested by Lamb et al; implying that doxycycline could be used to treat other types of cancers as well [48]. We have already shown that doxycycline can also induce apoptosis in diffuse large B-cell lymphoma cell lines [20]. Additionally another study independently suggested that doxycycline could be used as a therapy in other cancers [54]. Two additional studies have shown a potential role for doxycycline in the mammosphere-forming activity in breast cancers [55, 56]. Wan et al showed that doxycycline, in combination with aspirin, lysine, and mifepristone can prevent and treat cancer metastasis [57]. Qin et al showed that doxycycline suppressed proliferation and metastasis of lung cancer cells [58]. These promising results combined with our data, warrant further clinical trials on the efficacy of doxycycline in the treatment of these cancers. A clinical trial to study the efficacy and safety of doxycycline in patients with relapsed CTCL is underway [59]. Further study will identify the chemical components of doxycycline that make it a lymphoma killer.

## MATERIALS AND METHODS

### Tissue culture

MyLa cells were kindly provided by Andrew Feldman, M.D., from Mayo Clinic in Rochester, MN. Other cell lines were obtained through the American Tissue Culture Collection. H9, MyLa, HH, and primary T-cells were kept between 3×10^5^-1×10^6^cells/mL in RPMI media (Gibco) supplemented with 10% Fetal Bovine Serum (FBS, Gibco) and 1% Penicillin and Streptomycin (Gibco). MJ and Hut78 cell lines were grown in DMEM (Gibco) supplemented with 20% FBS and 1% Penicillin and Streptomycin. Cells were incubated at 37°C with 5% CO_2_.

### Doxycycline stock

Doxycycline hyclate (Sigma Aldrich,) stock solutions were made at 40mg/mL or 10mg/mL in water. A conversion factor of 93.76% was used to account for the hyclate. Samples were aliquoted into Eppendorf tubes and stored at −20°C protected from light. Stock solutions were diluted to final concentrations with medium.

### Trypan blue assays

Cells from pre-established cell lines were plated at 3×10^5^ cells/mL in 12 well plates and treated with doxycycline using the indicated concentrations. Primary cells were plated at 5×10^5^ cells/mL in 96 well round bottom plates. Cell viability was measured by adding a 1:1 trypan blue: cell sample solution onto a hemocytometer. Trypan blue was obtained from GIBCO.

### Mitochondrial and cytoplasmic extracts

Cytoplasmic extracts were separated from mitochondria using digitonin fractionation. Briefly, H9 cells were collected, washed with PBS, and resuspended in cytosolic lysis buffer (250mM Sucrose, 70mM KCl, 200ug/mL digitonin (Cayman), and protease inhibitor (Pierce Thermo Scientific). After confirming that >95% of cells had lysed by trypan blue, these were centrifuged at 1,000 x g for 5 minutes at 4°C. Supernatant was collected as the cytoplasmic fraction. The pellet was washed with PBS, and incubated for 10 minutes at 4°C in mitochondrial lysis buffer (50mM Tris HCl p.H. 7.4, 150mM NaCl, 2mM EDTA, 2mM EGTA, and 0.2% Triton X-100, 0.3% NP-40, plus protein inhibitor (Pierce Thermo Scientific)). The suspension was centrifuged at 10,000 x g for 10 minutes and the supernatant collected as the mitochondrial fraction.

### Whole cell lysates

Cells treated with doxycycline for specific times were removed from the incubator, collected on ice, pelleted and washed with cold (4°C) PBS. The pellet was resuspended in RIPA buffer (1X PBS, 1% Nonidet P-40, 0.5% Sodium Dodecyl Sulfate (SDS)) with 1:100 Halt Protease and Phosphatase inhibitor (Pierce Thermo Scientific)). Protein concentration was determined using BioRad's DC Protein Assay according to manufacturer's instructions. Samples were stored at −80°C.

### Western blot analysis

Protein lysates (20-25μg) were denatured using 1X Laemmli Buffer and run on 7.5, 10, or 16% SDS PAGE gels at 100V for about 2 hours. Proteins were then transferred onto a PDVF membrane (BioRad or Thermo Fisher Scientific) at 100V for 1 hour. Ponceau-S staining was done to verify transfer. Non-specific binding was blocked by incubating the membrane for at least one hour at room temperature or overnight at 4°C in 5% milk in TBS for non-phosphorylated proteins, or 5% Bovine serum albumin (BSA Fraction V, Fisher Scientific) in TBS for phosphorylated proteins. The membrane was then blotted with primary antibody diluted in 5% BSA or Milk in TBS supplemented with 0.1% Tween 20 (TBST). After an overnight incubation at 4°C, the membrane was washed with TBST and incubated in the secondary antibody for at least 2 hours at room temperature in 5% BSA or Milk in TBST. After washing out the secondary antibody in TBST, the membrane was exposed to BioRad's Clarity Western ECL substrate according to manufacturer's instructions. Signal was detected using UltraCruz Autoradiography Film (Santa Cruz Biotech). Antibodies for GAPDH and p-IκBα were obtained from Cell Signaling (Catalog # 14C10 and 14D4, respectively). BCL2α, Pro-caspase 3, PARP-1, and cytochrome c antibodies were obtained from Santa Cruz Biotech Catalog # SC-7382, SC-7148, SC-8007, and SC-271627, respectively.

### Apoptosis assay, flow cytometry

Cells were stained with PE-Annexin V (BD Pharmingen Apoptosis kit) and either 7AAD (BD) or PI (Research Organics). Selection of CD4+ in primary T-cells was performed concurrently with Annexin V stains using BioLegend's FITC tagged antibody

### PBMC isolation

Peripheral blood was obtained after subjects signed consent approved by the University of Rochester's Research Subject Review Board (RSRB No. 46521). Whole blood was collected in 6ml BD Vacutainer® plastic tubes with EDTA, and processed no longer than an hour after collection time. Samples remained at room temperature until they were processed. Whole blood was mixed with an equal volume of PBS and layered on top of Ficoll-Hypaque PLUS(GE) for isolation of peripheral blood mononuclear cells (PBMC) and centrifuged at 400 x g for 40 minutes with no break. PBMC was then washed three times with PBS and CD4+ cells were negatively selected using the CD4+ Human T-cell isolation kit (Miltenyi Biotech). Cells were incubated at a concentration of 5×10^5^ cells/mL in round bottom 96 well plates overnight and then treated with doxycycline.

### Reactive oxygen species assays

H9 Cells were treated with diphenyleneiodonium chloride (Cayman) at 0.1μg/mL, 0.5μg/mL and 1μg/mL or Catalase (Sigma Aldrich) at 200μg/mL, with doxycycline at 10μg/mL for 48 hours. Apoptosis assay using Annexin V and 7AAD was done as described above.

### Caspase 8 inhibition

Inhibitor Z-IETD-FMK (BD Pharmingen) was added at 2 and 20μg/mL, incubated at 37°C with 5% Carbon Dioxide for an hour and then treated with Doxycycline at 10 ug/mL for 48 hours. Annexin V and 7AAD stains were then done as described above.

### TNF stimulation

Cells were stimulated with human recombinant TNF at 10ng/mL for the indicated times at 37°C, collected on ice, washed with cold PBS once, and lysed for protein isolation. Lysates were stored at −80°C until further processing.
